# Using Fresh Frozen Plasma for Acute Airway Angioedema to Prevent Intubation in the Emergency Department: A Retrospective Cohort Study

**DOI:** 10.1155/2016/6091510

**Published:** 2016-02-03

**Authors:** Aya Saeb, Karen H. Hagglund, Christine T. Cigolle

**Affiliations:** ^1^Department of Internal Medicine, St. John Hospital & Medical Center, 22101 Moross Road, Detroit, MI 48236, USA; ^2^Geriatric Research Education and Clinical Center (GRECC), VA Ann Arbor Healthcare System, 2215 Fuller Road 11 G, Ann Arbor, MI 48105, USA; ^3^Department of Internal Medicine, University of Michigan, 3110 Taubman Center, SPC 5368, 1500 East Medical Center Drive, Ann Arbor, MI 48109, USA; ^4^Department of Family Medicine, University of Michigan, 1150 W. Medical Center Drive, M7300 Med Sci I, SPC 5625, Ann Arbor, MI 48109, USA

## Abstract

*Background*. Angioedema (AE) is a common condition which can be complicated by laryngeal edema, having up to 40% mortality. Although sporadic case reports attest to the benefits of fresh frozen plasma (FFP) in treating severe acute bouts of AE, little evidence-based support for this practice is available at present.* Study Objectives*. To compare the frequency, duration of intubation, and length of intensive care unit (ICU) stay in patients with acute airway AE, with and without the use of FFP.* Methods*. A retrospective cohort study was conducted, investigating adults admitted to large community hospital ICU with a diagnosis of AE during the years of 2007–2012. Altogether, 128 charts were reviewed for demographics, comorbidities, hospital courses, and outcomes. A total of 20 patients received FFP (108 did not).* Results*. Demographics and comorbidities did not differ by treatment group. However, nontreated controls did worse in terms of intubation frequency (60% versus 35%; *p* = 0.05) and ICU stay (3.5 days versus 1.5 days; *p* < 0.001). Group outcomes were otherwise similar.* Conclusion*. In an emergency department setting, the use of FFP should be considered in managing acute airway nonhereditary AE (refractory to steroid, antihistamine, and epinephrine). Larger prospective, better controlled studies are needed to devise appropriate treatment guidelines.

## 1. Introduction

Angioedema (AE), defined as self-limited, localized swelling, can manifest as an acute attack of asymmetric, nonpruritic, nonpitting subcutaneous or submucosal edema [1]. The reported prevalence varies, depending on etiology, population studied, and method of study (self-reported versus medically diagnosed). The World Allergy Organization (WAO) estimates that up to 20% of individuals will experience urticaria or AE at least once in their lifetime [2, 3]. In related studies, 5%–15% of patients with AE developed obstructive laryngeal edema [4], which is the leading cause of death. The latter carries a mortality rate of 25%–40% [1].

For emergency physicians, evaluation and management of patients who present with acute airway AE may be challenging. This disorder can be caused by a wide variety of immunologic and nonimmunologic mechanisms. Not surprisingly, there are a number of classification systems of AE in the medical literature. For the clinician, we found that dividing AE into two broad categories, histamine-mediated (also mast cell-mediated/allergic) and bradykinin-mediated, is the most clinically relevant and practical in guiding the management approach. When assessing acute AE affecting the airway, the emergency physician must rely on the patient's history and physical examination. With the exception of hereditary AE (HAE), laboratory studies such as serum levels of Complement 4, tryptase levels, C1 inhibitor protein, and the C1 inhibitor functional activity have been shown to have no utility in the evaluation of acute AE especially in the acute setting [1]. Most common cause of acute AE is thought to be histamine-mediated and is frequently responsive to antihistamine, epinephrine, and steroids; however, refractory cases may be secondary to bradykinin [5].

Fresh frozen plasma (FFP) is one treatment option for acute bradykinin-induced AE with airway compromise. The first report of its successful use was in hereditary AE in 1969 [6]. Subsequent case reports have demonstrated the effectiveness of FFP (given within 45 minutes to 12 hours) for both HAE and non-HAE [7, 8]. However, no randomized clinical trials have investigated the use of FFP for either condition [7]. Likewise, there are no studies that examine the frequency of intubation or the length of stay in intensive care unit (ICU) settings after administration of FFP for airway AE.

Recent literature demonstrates that FFP is effective and can be used if C1 esterase inhibitor (a recently approved treatment for HAE by the US Food and Drug Administration in 2009) is not available (e.g., due to cost) or if initial treatment does not work (i.e., steroid, antihistamine, and epinephrine) (strength of evidence, Level III Grade B) [9]. However, based on a large survey of physicians in the United States in 2011, only 34.9% use FFP in acute episodes of AE [10].

**Figure 1:**
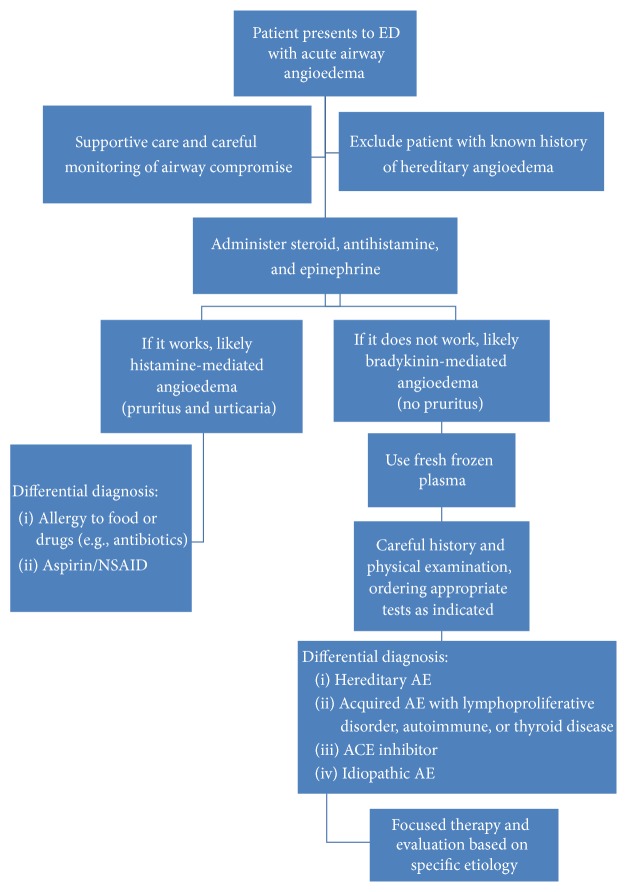
Management algorithm.

Given the challenge facing emergency physicians managing life-threatening acute AE with airway compromise, we investigated the use of FFP to examine its ability to prevent intubation and shorten the course of airway angioedema and hence ICU stays. After reviewing the comprehensive differential diagnosis for AE acute attacks, we devised our own simplified algorithm to illustrate the decision points for the emergency physician in managing airway angioedema and the place of FFP as an intervention (Figure 1). Proper management includes early recognition and aggressive airway management. For some etiologies, however, both treatment and patient disposition remain controversial [9].

Of note, our investigation focused on patients presenting with idiopathic angioedema. These episodes, those having an unidentified etiology (possibly histamine-mediated, initial attacks of hereditary or acquired angioedema) or those linked with angiotensin-converting enzyme (ACE) inhibitors use, are of interest, given the limited evidence base to guide management and the lack of any treatments approved specifically for these conditions by the US Food and Drug Administration.

We conducted a retrospective cohort study, to determine the frequency of intubation and its duration in patients who presented to our emergency department (ED) with acute AE involving the airway which required ICU admission, comparing patients with airway AE treated with FFP to those not treated with FFP (cases and controls, resp.). We also compared the length of stay in ICU settings for both groups, examining whether FFP administration shortens ICU stays in severe, acute attacks of AE. We hypothesized that using FFP in an acute AE attack with airway compromise can prevent or shorten intubation and lead to shortened ICU stays.

## 2. Methods

### 2.1. Design

A keyword search of PubMed (i.e., angioedema, intensive care unit, fresh frozen plasma, and intubation) identified no studies comparing the frequency of intubation, duration of intubation, or the length of ICU stays in AE cases with airway involvement managed with or without FFP.

We conducted a retrospective cohort study of all adults admitted to the ICU (medical and surgical) in a community teaching hospital in Detroit, MI (772 beds) with a diagnosis of AE during the years of 2007–2012. The scope was confined to chart review; therefore signed patient consent documents were not required. Once St. John Hospital and Medical Center Institutional Review Board approval was granted, all data obtained via electronic medical records and paper charts of ICU/hospital stays were handled in Health Insurance Portability and Accountability Act, compliant manner. The inclusion criteria were as follows:

1. Age ≥18 years.
2. Admission to medical or surgical ICU.
3. Primary diagnosis of AE (indicated by ICD-9 code) and airway involvement (indicated by documented history and physical exam). (The subset of patients taking ACE inhibitors was of particular interest.)

The exclusion criteria included the following:

1. Food allergy (if specified as a cause of AE).
2. Intubation unrelated to airway AE.
3. ICU admission for other indications (not AE primarily).
4. Known history of HAE. (Patients with histories of HAE were excluded on the grounds that their diagnoses were already established and therefore prophylactic treatments were likely in use, perhaps with the access to other recently FDA-approved targeted therapies.)

### 2.2. Study Population and Main Measures

Paper and electronic medical records/charts were reviewed retrospectively. Dependent variables included intubation frequency, duration (in hours), site of intubation (ED, ICU, or hospital floor), performing physician (emergency physician versus anesthesiologist or otolaryngologist), and route (oral versus nasal), as well as the need for cricothyroidotomy or tracheostomy, ICU stay (in days), and patient outcome/disposition (discharged home, transitioned to extended care facility, or deceased). Important covariates included patient demographics (age, gender, and race), admission and discharge dates, admission source (ED, extended care facility, or direct admission from clinic), comorbidities (diabetes mellitus, hypertension, congestive heart failure, asthma, chronic obstructive pulmonary disease, obstructive sleep apnea, chronic kidney disease, and others), ACE inhibitor use, known specific allergies (food or antibiotics) by history, individual or family history of AE, and history of prior intubation.

We conducted a retrospective cohort study given the anticipated limited use of FFP for airway AE in the ED settings plus our interest in measuring multiple outcomes. The rationale behind the ED or ICU physician's choice of FFP was not always indicated in the medical chart. It is possible that one explanation is a lack of knowledge about the role of FFP as a treatment of airway AE; this lack of knowledge could be a major confounder in our analyses. Data were collected by the primary author from multiple parts of the electronic and paper medical records (e.g., physician progress note, respiratory therapist note, etc.) to ascertain consistency and avoid recall/information bias.

### 2.3. Statistical Analysis

Given that most of the studies evaluating FFP for airway AE are descriptive and that there is a lack of data in prior studies on the frequency of intubation, a reference rate was not available. We conservatively estimated that 90% of patients evaluated in the ED for acute AE with airway compromise and not treated with FFP would require intubation; in contrast, 70% of those treated with FFP would require intubation. Given this effect size, 62 patients were needed for each group (FFP versus no FFP) for 80% power and alpha = 0.05.

By chart review, we had 160 charts but only 128 patients met the inclusion criteria. Twenty patients had received FFP, with the remainder (*n* = 108) serving as controls. Despite being underpowered, statistically significant results were achieved.

The frequencies and other descriptive statistics were calculated, and associations between categorical variables were assessed via the chi-square test. Between-group differences in continuous variables were measured using *t*-tests for independent groups. Standard software was used (SPSS v12.0; SPSS Inc., Chicago, IL, USA), with statistical significance set at *p* < 0.05.

## 3. Results

Out of 160 charts only 128 patients met our inclusion criteria. Mean ± SD patient age was 61 ± 14.8 years. Just over half of the cohort was female, and 84% were African-American. Demographics, comorbidities, and medical histories did not differ significantly by group (*p* > 0.05) (Table 1).

Hypertension was prevalent in both FFP-treated (95%) and control groups (90%), and ACE inhibitors were frequently used (90% and 85%, resp.). Most patients had documented allergies to two or more medications. Past histories of AE were documented in 30% of FFP-treated patients, compared with 24% of controls. Subanalysis of each comorbidity indicated no measured effect on either primary or secondary outcomes.

Intubation was done in 35% of the FFP-treated patients, compared with 60% of controls (*p* = 0.050). FFP recipients were intubated for 60 hours, as opposed to 97 hours in controls (*p* = 0.45) (Table 2). Surprisingly, most intubations were performed by an anesthesiologist or an otolaryngology surgeon (FFP, 100%; controls, 86%). Cricothyroidotomy or tracheostomy was performed in small numbers of patients of both groups, with no statistically significant difference (*p* = 0.19 and *p* = 0.34, resp.).

**Figure 2:**
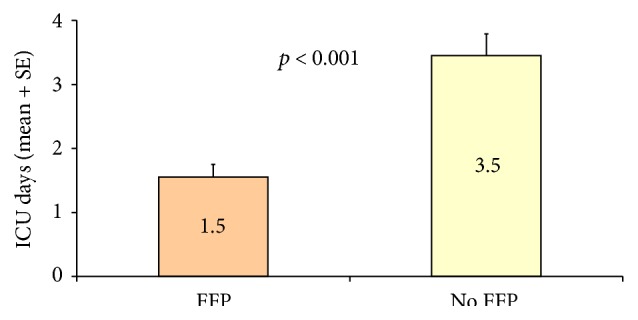
ICU stays differed significantly by group. FFP, fresh frozen plasma; ICU, intensive care unit; SE, standard error.

**Figure 3:**
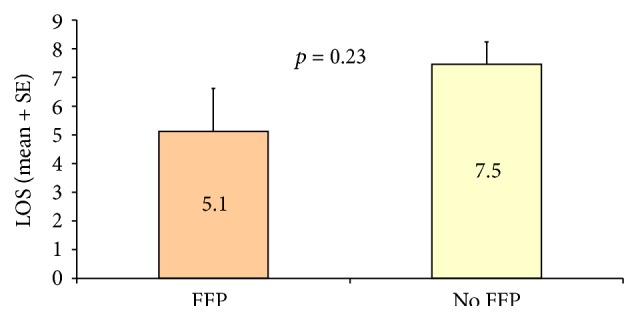
Difference in total hospital stays by group. FFP, fresh frozen plasma; LOS, length of stay; SE, standard error.

By comparison, FFP-treated patients had shorter ICU stays (1.5 days versus 3.5 days; *p* < 0.001). Although the extent of hospital stay differed by group, this difference was not statistically significant (FFP, 5 days; controls, 7.5 days; *p* = 0.23) (Figures 2 and 3).

**Figure 4:**
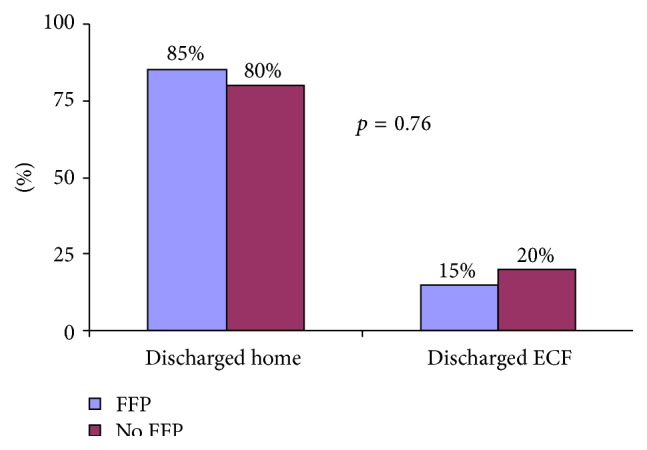
Differences in group outcomes did not reach statistical significance. FFP, fresh frozen plasma; ECF, extended care facility.

There were no differences in discharge dispositions between the groups (*p* = 0.76) (Figure 4). Overall, 78% returned home and 19% were released to an extended care facility (ECF).

## 4. Discussion

The goal of our study was to determine the frequency of intubation and ICU length of stay in patients presenting with acute (nonhereditary) AE and airway compromise who received FFP, comparing these findings with non-FFP-treated patients. To the best of our knowledge, this is the first paper to examine this relationship. Given the retrospective nature of the study, we acknowledge the limitation to ascertain the exact frequency or duration for both outcomes. However we were able to observe a reduction in the ICU length of stay among FFP-treated patients by comparison with non-FFP-treated patients. In addition, there was a reduction in the frequency of intubation that just missed statistical significance (*p* = 0.050).

The various AE syndromes all share the characteristic swelling and subcutaneous/submucosal tissue edema due to the release of vasoactive mediators, including histamine and bradykinin. We selectively studied patients with idiopathic AE (nonhereditary). As explained earlier, well-designed studies and clear management guidelines for this fairly common condition are lacking.

The role of bradykinin and bradykinin type 2 receptors in the development of AE is well documented and has prompted the discovery of new treatments for HAE [11, 12]. ACE inhibitors decrease bradykinin catabolism, which boosts plasma levels of bradykinin and exacerbates bradykinin-dependent variants of AE [8, 13]. The reported prevalence of AE associated with ACE inhibitors ranges from 0.1% to as high as 6% in some prospective clinical trials [11]. FFP works in bradykinin-induced AE by supplying C1-INH and angiotensin-converting enzyme (ACE) among others to breakdown the accumulated levels of bradykinin.

Only recently (2009) has the US Food and Drug Administration approved other modalities/formulations (plasma-derived human C1-INH, plasma kallikrein inhibitor, C1-INH concentrates, nanofiltered plasma-derived human C1-INH, and bradykinin B2 receptor antagonist) for treating HAE attacks only [10]. Off-label use of some of these agents such as bradykinin B2 receptor antagonist in ACE inhibitors-induced AE has been reported with successful results [14]. However, the cost and availability limit in-hospital usage of these agents.

A large survey of US physicians conducted between October 2009 and February 2010 indicated that strategies for managing HAE vary substantially, with physicians still relying chiefly on supportive therapy for acute attacks. About 75% of physicians in this survey were unfamiliar with new FDA-approved treatments for HAE. Possible barriers for the use of these newly approved agents include cost, insurance restrictions, and practicality of drug delivery (i.e., lack of self-administration programs) [10].

As such, we decided to explore the use of FFP for life-threatening bouts of airway AE, particularly in instances where etiologies were unclear or difficult to ascertain. FFP use in acute attacks has proved beneficial and may be a reasonable option if C1-INH is too costly or unavailable, or in patients refractory to traditional treatment (steroid, antihistamine, and epinephrine) [9]. There is a theoretical risk that FFP can aggravate symptoms of AE but review of literature from 1969–2003 has not revealed any report to demonstrate such risk [15].

Side effects of FFP include alloimmunization, anaphylactic or allergic reaction, infection transmission (viral, Creutzfeldt-Jakob disease), and intravascular volume overload. However, the related risks must be weighed carefully against the danger of a life-threatening airway compromise [16]. Plasma is also available as a solvent/detergent-treated product, which ostensibly lowers the comparative risk of viral transmission, but the data on solvent detergent-treated plasma are scant [17].

FFP dosing for AE has not been studied and generally is administered as in coagulation disorders, infusing 2 units of 200 mL each (10 mL/kg) [16]. If volume overload is an issue, then 10–15 mL per kg body weight is recommended instead, with careful monitoring of volume status and cardiopulmonary function [17].

### 4.1. Strengths

Our study was done in a community hospital, with the majority of the study sample having African-American ethnicity. This has important implications for the generalizability of the study's findings to minority populations. The majority of study participants were being treated with ACE-I for hypertension. None of these patients was found to have another etiology for their AE, suggesting that FFP might be considered in ACE-I induced AE. This is an important finding, due to the broad use of ACE-I for hypertension and other indications. Although the number of patients who received FFP did not reach the study's calculated target to achieve 80% power, we nonetheless achieved statistically significant differences for ICU length of stay, which might have important implications in reducing cost of inpatient stays and risk for iatrogenic complications.

### 4.2. Limitations

The major limitation of this investigation includes its retrospective approach with two related issues. The first one is the chart review with the following limitations: incomplete or missing data within the medical record, difficulty in interpreting or verifying documented information, and variability in the quality of documentation among health care personnel. The second one is the lack of control over unmeasured variables which can be potential confounders, such as patient level of severity, time limitation, availability of FFP, patient preferences, and treating physician knowledge about available treatment options for AE. We had no valid measures of disease severity.

The effectiveness of FFP as a therapeutic option for AE with airway compromise needs a better, well-controlled design to establish; however by comparing the two groups in the same cohort (cases and controls) in our study we were able to shed the light into statistical significant differences with our measured outcomes. The poor general awareness of available treatment options for AE [10] and the bewildering array of guidelines for AE variants made this study challenging to conduct.

## 5. Conclusions

In patients with acute idiopathic AE and airway compromise, we hypothesized based on our observation that using FFP (versus no FFP) shortened the ICU stay. To our knowledge, this is the first study to explore this relationship. It was our intent to shed light on a widely available, promising intervention that is suitable for use in ED settings, should conventional modalities (steroid, antihistamine, and epinephrine) fail. We also looked to bridge the gap in knowledge of ED physicians and other practitioners who are dealing with such a fairly common and life-threatening condition. A final clinical implication of the study would include simplifying and solidifying the ED practice when managing acute AE and airway compromise. We hope that our study will spur further prospective well-controlled studies on a larger scale to test our hypothesis and to generate treatment guidelines that would address both idiopathic and ACE-I induced AE.

## Figures and Tables

**Table 1:** Patient characteristics.

Parameter	FFP (*n* = 20)	No FFP (*n* = 108)	*p* value
Age, years, mean (SD)	62.9 (11.1)	61.2 (15.4)	0.64
Male gender, *n* (%)	10 (50)	50 (46)	0.81
Race, *n* (%)			1.00
Caucasian	3 (15)	18 (17)	
African-American	17 (85)	89 (83)	
Admission type, *n* (%)			—
ED	19 (95)	98 (90)	
ECF	1 (5)	5 (5)	
Direct	0 (0)	5 (5)	
Comorbidities, *n* (%)			
Diabetes mellitus	9 (45)	28 (26)	0.11
Hypertension	19 (95)	97 (90)	0.69
CHF	2 (10)	7 (7)	0.63
Asthma	1 (5)	12 (11)	0.69
COPD	6 (30)	26 (24)	0.58
OSA	4 (20)	8 (7)	0.09
CKD	1 (5)	20 (19)	0.19
Cancer	0 (0)	10 (9)	0.36
History of allergies, *n* (%)	20 (100)	106 (98)	1.00
Number of allergies, mean (SD)	2.0 (1.0)	2.4 (1.6)	0.34
History of ACE-I, *n* (%)	18 (90)	92 (85)	0.74
History of angioedema, *n* (%)	6 (30)	26 (24)	0.58
History of intubation, *n* (%)	3 (15)	4 (4)	0.08

ACE-I, ACE inhibitor; CHF, congestive heart failure; CKD, chronic kidney disease; COPD, chronic obstructive pulmonary disease; ECF, extended care facility; ED, emergency department; FFP, fresh frozen plasma; OSA, obstructive sleep apnea; SD, standard deviation.

**Table 2:** Intubation characteristics.

Parameter	FFP (*n* = 20)	No FFP (*n* = 108)	*p* value
Prevalence, *n* (%)	7 (35)	65 (60)	0.05
Duration, hours, mean (SD)	60.3 (38.2)	97.1 (126.9)	0.45
Type, *n* (%)			1.00
PO	5 (83)	51 (81)	
Nasal	1 (17)	12 (19)	
Location, *n* (%)			—
ED	3 (43)	45 (69)	
ICU	1 (14)	3 (5)	
Floor	0 (0)	9 (14)	
OR	3 (43)	8 (12)	
Procedure administrator, *n* (%)			0.59
ED physician	0 (0)	9 (14)	
Anesthesiologist/ENT	7 (100)	56 (86)	
Procedures, *n* (%)			
Cricothyroidotomy	1 (14)	1 (2)	0.19
Tracheostomy	1 (14)	3 (5)	0.34

ED, emergency department; ENT, ear, nose, and throat surgeon; FFP, fresh frozen plasma; ICU, intensive care unit; OR, operating room; PO, per-oral; SD, standard deviation.
